# Automated image analysis in the study of lymphocyte subpopulation in eosinophilic oesophagitis

**DOI:** 10.1186/1746-1596-9-S1-S7

**Published:** 2014-12-19

**Authors:** Marcial García-Rojo, Joaquín Rodríguez Sánchez, Eva de la Santa, Elena Durán, José Luis Ruiz, Antonio Silva, Francisco Javier Rubio, Antonio M Rodríguez, Blas Meléndez, Lucía González, Bartolomé López-Viedma

**Affiliations:** 1 Department of Pathology. Hospital de Jerez de la Frontera, 11407 Cádiz, Spain; 2 Department of Gastroenterology. Hospital General Universitario de Ciudad Real, 13005, Ciudad Real, Spain; 3 Department of Pathology. Hospital General Universitario de Ciudad Real, 13005, Ciudad Real, Spain

## Abstract

**Background:**

Eosinophilic oesophagitis (EoE) is characterized by the presence of eosinophils in oesophageal mucosa. Other inflammatory cells, mainly lymphocytes, dendritic cells, and mast cells may also play an important role in this disease. The aim of this study is to compare the inflammatory pattern of the mucosa between EoE and gastro-oesophageal reflux disease (GERD), using automatic image analysis in digital slides, and to assess treatment response after elimination diet and food challenge test.

**Methods:**

From 2010 to 2013, 35 oesophageal biopsies from EoE and GERD patients were randomly selected. In six EoE biopsies, patients had been treated with selective food exclusion diet. Immunohistochemical study with CD3, CD20, CD4, and CD8 for lymphocyte populations, CD1a for dendritic cells, and CD117/c-kit for mast cells was performed. Slides were scanned using Leica Aperio Scanscope XT with 40× magnification. Immunohistochemical expression was quantified in 245 immunohistochemistry digital slides with Leica Aperio positive pixel count algorithm using two different approaches: whole slide analysis versus selection of a 2 mm^2 ^hot spot area.

**Results:**

Average eosinophil cell count was significantly higher (p < 0.001) in the first biopsy of EoE patients before treatment (30.75 eosinophils per high power field - HPF) than in GERD patients (0.85 eosinophils/HPF) or in EoE patients after treatment with elimination diet (1.60 eosinophils/HPF). In the immunohistochemical study, manual count and automatic image analysis showed a significant increase in the number of CD3 and CD8 cells in EoE patients, compared with GERD patients. However, the increase of CD117/c-kit was only statistically significant when manual counting procedures were used. CD20 positive cell count also showed a non-statistically significant tendency to reduce after elimination diet treatment.

Manual eosinophil count correlated much better with CD3 and CD8 count using hot spot approach than with a whole slide approach.

**Conclusions:**

Positive pixel count algorithm can be a useful tool to quantify the immunohistochemical expression of inflammatory cells in the diagnosis and follow up of eosinophilic oesophagitis.

## Background

Automatic image analysis can be very useful in the objective assessment of cell subpopulations. This can be extremely important in the research of diseases characterized by the presence of specific cell populations in routine histopathological sections or in immunohistochemical tests.

Eosinophilic oesophagitis is characterized by the presence of eosinophils in oesophageal mucosa [1]. Since normal mucosa does not show these cells, their presence is always pathological, and it is usually associated with gastroesophageal reflux disease (GERD), proton pump inhibitors responsive oesophageal eosinophilia, and eosinophilic oesophagitis (EoE).

The American College of Gastroenterologists includes as minor criteria for diagnosis of eosinophilic oesophagitis the increase in the number of lymphocytes and mast cell in oesophageal mucosa [2].

Some previous studies did not find significant differences in lymphocytes count (FoxP3, CD8, and CD4) between EoE and GERD [3].

After pharmacological or diet treatment, eosinophils may disappear from the oesophageal mucosa [4]. However, in eosinophilic oesophagitis some inflammatory response may be still be present, even after the decrease in the number or the absence of eosinophils.

The aim of this study is to compare the inflammatory pattern of EoE with GERD in the oesophageal mucosa, and to assess treatment response after elimination diet (legume, egg, milk, etc.), using automatic image analysis in digital slides of oesophageal endoscopic biopsies.

## Methods

This is an analytic observational retrospective case-control study. From 2010 to 2013, 35 oesophageal endoscopic biopsies from 20 patients were randomly selected from pathology department information database. From these, 10 patients (14 biopsies) were diagnosed as GERD and 10 patients (21 biopsies) were diagnosed of EoE, matched by age and sex for the same period of time. In six EoE biopsies, patients from EoE had been treated with selective food exclusion diet during follow up (from 6 months to 48 months).

Main reasons for exclusion in this study were biopsies with a significant amount of stroma or lack of enough tissue left in the paraffin block to perform a complete immunohistochemical study.

Oesophageal biopsies were processed after 24 h fixation in 10% formalin. Routine Haematoxylin and Eosin (H&E) stain and conventional immunohistochemistry were performed.

Inflammatory cell count was performed using monoclonal antibodies to detect lymphocyte subpopulations (CD3 [clone F7.2.38], CD20 [clone L26], CD4 [clone 4B12], and CD8 [clone C8/144B]), CD1a [clone O10] dendritic cells, and mast cells (CD117/c-kit [polyclonal]). All antibodies were from Dako (Denmark). Dako EnVision™ FLEX was used as visualization system, in a Dako Autostainer Plus. 3,3'-diaminobenzidine (DAB) was used as chromogen.

Most patients with a diagnosis of GERD had mainly one single biopsy from distal oesophagus. Patients with a confirmed or suspected diagnosis of EoE usually had 3 biopsies taken in each endoscopy session (proximal, medial, and distal oesophagus).

From each biopsy, 8 slides were completely scanned (including one H&E and the six immunohistochemical markers). A total of 280 slides were scanned using Leica Aperio Scanscope XT with 40× magnification, SVS (Aperio TIFF) as format file and JPEG2000 compression quality of 70.

Eosinophils counts were performed using high power fields (HPF) at 40× magnification in Aperio Imagescope digital slide viewer v12.0.0.5039. Two pathologists reviewed each slide and manually selected the areas with largest number of eosinophils. The average of the values obtained from each pathologist was recorded. In case a significant discrepancy was found between the values obtained by the two pathologists, a consensus was achieved.

Immunohistochemical expression of CD3, CD20, CD4, CD8, CD1a, and CD117/c-kit markers was quantified in 245 digital slides with Leica Aperio positive pixel count algorithm version 9.1, as previously described [5]. Two approaches were used with this algorithm: whole slide and "hot spot".

Collected variables in H&E stain were: Number of eosinophils per x40 high-magnification field under the conventional microscope, number of eosinophils per square millimetre using Aperio Imagescope, eosinophil cell degranulation (0-3), eosinophil microabscess formation (0-3), epithelial desquamation (0-3), epithelial basal hyperplasia (0-3), spongiosis (0-3), and lamina propria fibrosis (0-3).

For each immunohistochemical marker, the following variables were measured in the digital slide, using Leica Aperio Imagescope digital slide viewer: Number of positive cells per square millimetre (manual count), whole slide Noise to Signal Ratio (NSR), hot spot NSR, and distribution of positive cells.

**Figure 1:**
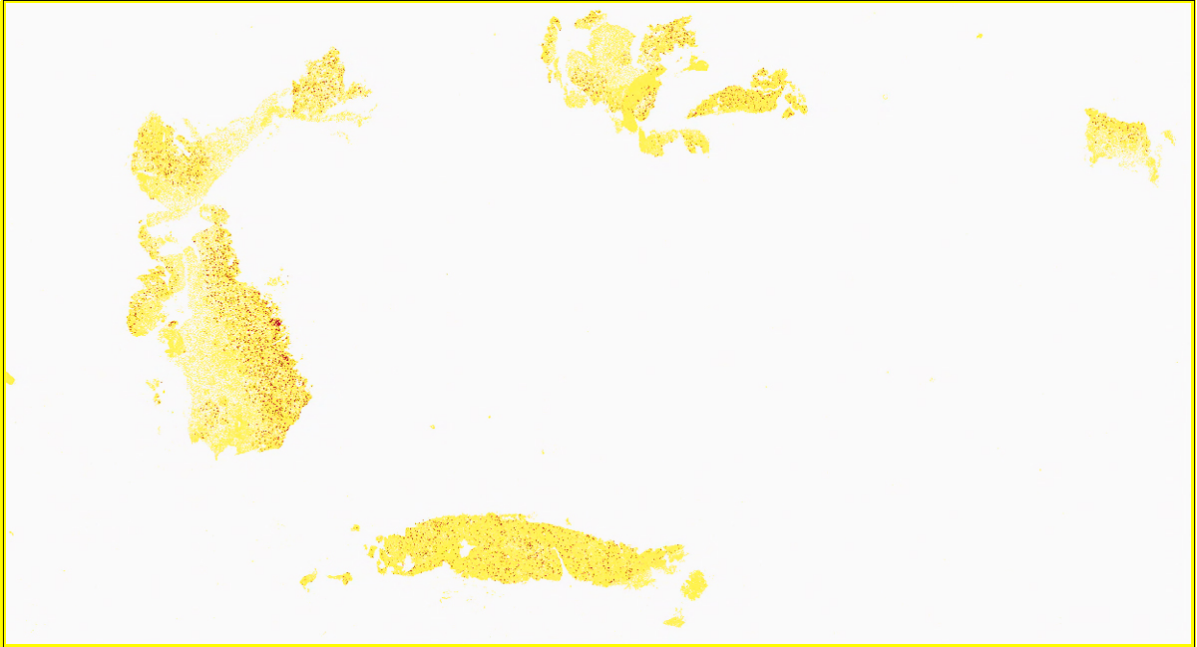
**Whole slide automatic analysis**. Mark-up image obtained from Leica Aperio positive pixel count to detect CD8 positive cells using the whole slide method.

Whole slide NSR is calculated selecting all the oesophageal tissue present in the digital slide. NSR is the ratio of strong positive pixels (DAB) stained with to total (tissue) pixels. Obtained figures are multiplied by 100 to obtain the percentage of tissue that expresses the antibody. A mark-up image is also generated in the whole slide analysis (Figure 1).

**Figure 2:**
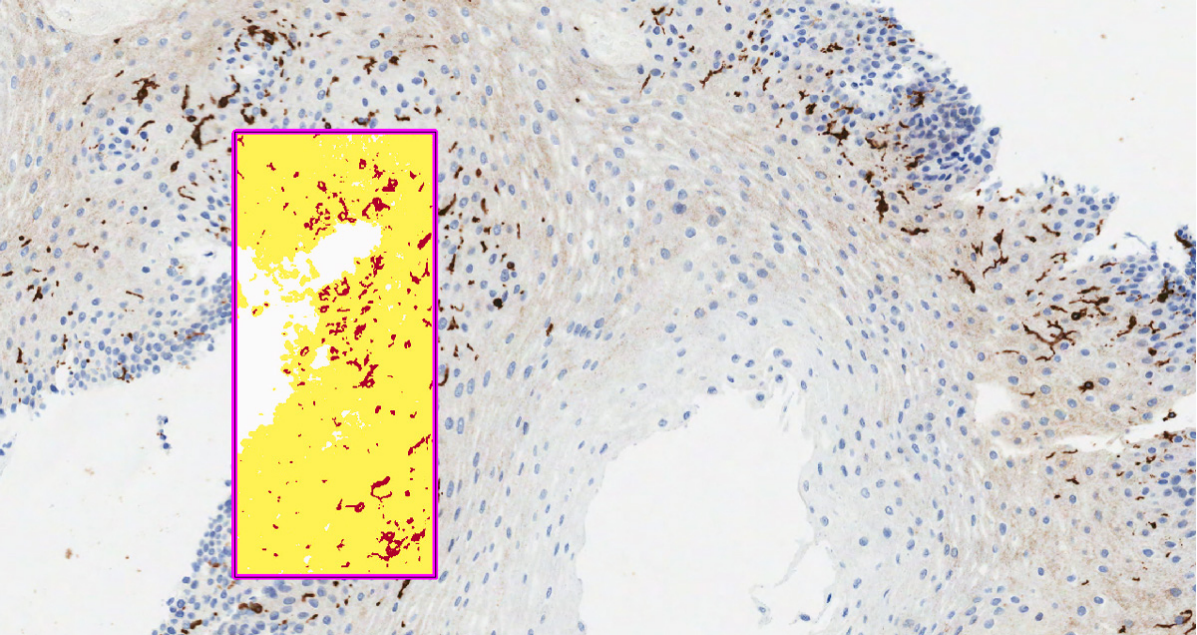
**Hot spot automatic analysis**. Leica Aperio positive pixel count to detect CD1a positive cells using a hot spot approach.

Using obtained image mask from the whole slide analysis, hot spot NSR was calculated by selecting a 2 mm^2 ^area ("hot spot") with higher concentration of positive cells, in order to perform a second analysis (Figure 2). Stromal areas were skipped in this hot spot analysis.

Immunohistochemical marker distribution options were: absence, only in stroma, inferior one third of the epithelium, inferior two thirds of the epithelium, and diffuse.

The input parameters used to find out the percentage of tissue stained with the immunohistochemical marker are collected in table 1.

**Table 1:** Positive pixel count algorithm input parameters.

View Width: 1000 View Height: 1000 Overlap Size: 0 Image Zoom: 1 Mark-up Compression Type: Same as processed image Compression Quality: 30. Classifier Neighbourhood: 0 Classifier: None Class List: None Hue Value: 0.1 Hue Width: 0.5 Colour Saturation Threshold: 0.5 Iwp (High): 230 Iwp (Low) = Ip (High): 70 Ip (Low) = Isp (High): 70 Isp (Low): 0 Inp (High): -1

### Statistical analysis

Differences in percentages and cell counts were calculated with the chi-square and Student's t-test. When using image analysis, immunostaining score values for each protein were expressed as a percentage. Correlation between score values was calculated by using the Pearson's r correlation test. Comparison of immunostaining values between groups was made with the Mann-Whitney or Kruskall-Wallis tests. Statistical results were corrected applying Bonferroni's correction. Univariate and multivariate analyses were performed. SPSS for windows version 16.0 software (Chicago, IL, USA) was used for all calculations. Statistical significance was defined as p < 0.05.

## Results

### Histopathological findings with conventional stains (H&E)

Regardless of the counting method used, average eosinophil cell count was significantly higher (p < 0.001) in the first biopsy of EoE patients before treatment than in GERD patients, both in eosinophils per 40× field (30.75 ± 14.83, range 4 - 54 vs 0.85 ± 1.63, range 0 - 4) and in eosinophils per mm^2 ^(88 ± 58.95, range 6 - 189 vs 1.69 ± 3.47, range 0 - 11) (Table 2).

**Table 2:** Number of eosinophils in oesophageal mucosa.

Disease	Number of biopsies	Mean Eos/HPF	Men Eos/mm^2^
Eosinophilic oesophagitis	21	23.71 +/- 18.23	67,81 +/- 63,13

Gastroesophageal reflux disease	14	0.85 +/- 1.62	1,69 +/- 3,47

Descriptive statistics.

After treatment with specific food elimination in EoE patients, eosinophils count dropped to 1.60 [range 0 - 6] eosinophils per 40× field and 4.62 [range 0 - 16] eosinophils per mm^2^.

Epithelial spongiosis of the oesophageal epithelial was present only in 4 of the 10 GERD patients. In contrast to EoE, the rest of the examined histopathological findings (eosinophil cell degranulation, eosinophil microabscess formation, epithelial desquamation, and epithelial basal hyperplasia) were not present in biopsies from GERD patients.

A slight fibrosis of the lamina propria of the mucosa was present in 3 of the 5 GERD patients in whom it could be evaluated. Lamina propria fibrosis was consistently found in all eight EoE patients (with or without treatment) in whom lamina propria could be evaluated.

### Immunohistochemical markers

CD20 and CD1a did not show significant differences between GERD and EoE patients, using manual counting method. Using automatic counting methods significant differences were found in CD4 between GERD and EoE before treatment that were not observed using manual count method (Table 3).

**Table 3:** Average values for immunohistochemical markers.

Marker	GERD	EoE pre-treatment (%)	EoE post-treatment (%)
CD20			

Manual count	3,61	7,50 (n.s.)	2,66 (n.s.)

Whole slide %	0.05	0.11 (n.s.)	0.05 (n.s.)

Hot spot %	0.37	1.15 (n.s.)	0.18 (n.s.)

CD3			

Manual count	30.57	58.87 (p = 0.004)	39.40 (n.s.)

Whole slide %	1.23	2.56 (p = 0.04)	1.31 (n.s.)

Hot spot %	2.10	5.71 (p = 0.01)	2.76 (n.s.)

CD4			

Manual count	17.15	20.10 (n.s.)	26.33 (n.s.)

Whole slide %	0.25	0.30 (n.s.)	0.90 (p < 0.05)

Hot spot %	0.20	0.99 (p = 0.01)	1.79 (p = 0.05)

CD8			

Manual count	24.69	57.90 (p = 0.01)	47.00 (n.s.)

Whole slide %	0.62	2.09 (p = 0.02)	1.03 (n.s.)

Hot spot %	1.79	4.37 (n.s., p = 0.06)	2.99 (n.s.)

CD1a			

Manual count	19.23	25.70 (n.s.)	19.00 (n.s.)

Whole slide %	1.35	0.99 (n.s.)	1.13 (n.s.)

Hot spot %	2.74	2.01 (n.s.)	2.23 (n.s.)

CD117/c-kit			

Manual count	5.62	12.64 (p < 0.05)	5.00 (p < 0.05)

Whole slide %	0.08	0.15 (n.s.)	0.06 (n.s.)

Hot spot %	0.26	0.45 (n.s.)	0.22 (n.s.)

n.s. Statistically, not significant

Using manual count, patients with EoE before treatment showed a significant larger number of CD3 cells (58.87 ± 25.78 vs 30.57 ± 15.72; p = 0.004), CD8 (57.17 ± 26.78 vs 24.69 ± 11.98; p = 0.002), and CD117/c-kit (12.64 ±7.03 vs 5.61 ± 4.57; p = 0.005), compared with GERD patients.

The statistical significance in CD3 and CD8 counts between EoE and GERD patients was also shown using automatic image analysis, both with whole slide and hot spot approaches, but not for CD117/c-kit. CD4 count was significantly higher in EoE biopsies before treatment than in GERD biopsies, only when hot spot automatic image analysis method was used.

Biopsies from EoE patients treated with exclusion diet showed a significant increase in CD4 using automatic counting method compared with EoE patients before treatment. Conversely, CD20 counts from the biopsies taken after treatment were lower than before treatment, but differences were not statistically significant with any of the three used methods (manual, whole slide and hot spot).

### Correlation study between eosinophil count and immunohistochemical markers

**Figure 3:**
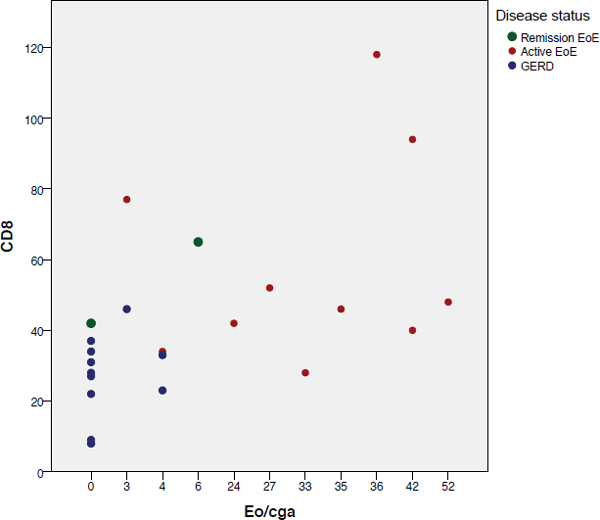
**Eosinophils and CD8 correlation**. CD8 and CD117/c-kit (not shown) showed and excellent correlation with manual eosinophils count (Eo/cga).

Manual eosinophil count correlated positively with most immunohistochemical markers in this study (CD20 r = 0.37, p = 0.03; CD3 r = 0.57, p = 0.01; CD8 r = 0.51, p = 0.01; CD117/c-kit r = 0.57, p = 0,001), except for CD4 and CD1a (Figure 3).

Considering CD3 and CD8 counts, positive pixel count algorithm has a positive predictive value of 98% and a negative predictive value of 83% in the diagnosis of eosinophilic oesophagitis.

When different counting methods are compared, manual eosinophil count correlated much better with CD3 and CD8 count using hot spot approach than with a whole slide approach.

## Discussion

Regarding eosinophils manual counting methods (eosinophils per HPF 40× field, and eosinophils per mm^2^), similar results were obtained with both procedures.

Since conventional methods to evaluate eosinophilic oesophagitis are prone to subjective interpretation by pathologists, in order to evaluate inflammatory pattern with objective criteria, we performed automatic inflammatory cell count using Leica Aperio positive pixel count, on immunohistochemical slides to detect mast cells, dendritic cells, and lymphocyte subpopulations. In our study, positive pixel count algorithm applied to whole slide was useful to distinguish EoE from GERD biopsies.

An automatic analysis of CD3, CD4 and CD8 markers can be used to differentiate between EoE from GERD patients. Most EoE biopsies showed more than 2% of the whole tissue area (excluding stroma) stained with CD3 or CD8, whereas GERD patients generally showed 1%, or less, of the whole tissue area stained with those markers.

Only hot spot automatic analysis was able to show significant difference between EoE and GERD in CD4 positive cell count. CD4 cells are important in the inflammatory response of both EoE and GERD patients. An automatic analysis of this marker was also able to show that there is a significant increase of CD4 cells on EoE biopsies after treatment, compared to biopsies before diet. The reasons for this increment in T-cell lymphocytes after treatment are not well explained in the literature.

Automatic image analysis, both with whole slide and hot spot approaches, was not able to show the statistical significance between EoE and GERD patients in CD117/c-kit analysis. This may be due to the small number of mast cells present in EoE patient (usually, less than 20 per mm^2^) and the small number of patients include in this series.

In the literature, positive pixel count method has shown to be effective in quantifying cells, also in lymphocytic subpopulations [6]. This algorithm is generally used to quantify the amount of a stain present in a digital slide. Using brown from 3,3'-diaminobenzidine as colour specification, the algorithm counts the number and intensity in the assigned intensity range, and the weak pixels parameters were used to adjust the total amount of tissue present in the digital slide.

In this study, differences in manual and automatic counting procedures are difficult to interpret due to the low number of patients included in this series. We propose that automatic procedures may help to detect small differences - like in CD4 expression-that manual counts are not able to detect due to subjective variations, mainly in small series of patients.

In our study, mark-up image generated in the whole slide analysis allowed for an easier detection of hot spot, defined as areas in which an immunohistochemical marker is particularly prevalent. This can be of paramount importance when tissue heterogeneity is significant [7].

In general, after diet exclusion treatment, oesophageal biopsies show a lower number of CD20 positive lymphocytes and an increased number of CD3, CD4 and CD8 cells compared with biopsied from non-treated EOE patients, although future studies with larger number of biopsies from treated patients are necessary to confirm this findings.

## Conclusions

A simple and free tool, such as Leica Aperio Positive Pixel Count, can be useful to quantify area and intensities of positive and negative immunohistochemistry markers. Automatic CD3, CD4 and CD8 staining quantification in whole slides was useful to distinguish EoE from GERD biopsies, and it may be at least as useful as manual counts in the diagnosis and follow-up of eosinophilic oesophagitis.

## Abbreviations

DAB: Diaminobenzidine; EoE: Eosinophilic oesophagitis; GERD: Gastroesophageal reflux disease; H&E: Haematoxilin and eosin; HPF: High power field; NSR: Noise to signal ratio; GERD: Gastroesophageal reflux disease

## Competing interests

The authors declare that they have no competing interests.

## Authors' contributions

Conceived and designed the experiments: MGR, JR. Performed the experiments: MGR, JR, ES, BLV. Analyzed the data: MGR, JR. Contributed reagents, materials, analysis, tools: MGR, JR. Wrote the paper: MGR, JR, ES, ED, JLR, AS, FJR, AMR, BM, LG, BLV.

## Notes

Publication of this supplement has been funded by 12th European Congress on Digital Pathology. This article has been published as part of *Diagnostic Pathology *Volume 9 Supplement 1, 2014: Selected articles from the 12th European Congress on Digital Pathology. The full contents of the supplement are available online at http://www.diagnosticpathology.org/supplements/9/S1
